# Efficacy and safety of direct oral anticoagulants in pediatric patients: a systematic review and meta-analysis

**DOI:** 10.3389/fpubh.2026.1787819

**Published:** 2026-04-07

**Authors:** Siyi You, Yan Gan, Xuemei Sun, Ying Tang, Junjie Ying, Dezhi Mu

**Affiliations:** 1 Department of Pediatrics, West China Second University Hospital, Sichuan University, Chengdu, China; 2 Department of Ultrasonography, West China Second University Hospital, Sichuan University, Chengdu, China; 3 Key Laboratory of Birth Defects and Related Diseases of Women and Children, Ministry of Education, NHC Key Laboratory of Chronobiology, Sichuan University, Chengdu, China

**Keywords:** anticoagulants, child, meta-analysis, randomized controlled trials, safety, thromboembolism

## Abstract

**Background:**

Thromboembolic events (TE) are increasingly recognized in pediatric patients, necessitating optimal antithrombotic therapy. While direct oral anticoagulants (DOACs) offer potential advantages over standard of care (SOC) such as heparin, comprehensive evidence regarding their use for treatment and prophylaxis remains limited. Therefore, we performed a systematic review and meta-analysis to assess the efficacy and safety of these two classes of anticoagulants.

**Methods:**

We searched PubMed, Embase, Web of Science, and ClinicalTrials.gov up to November 27, 2025, for relevant randomized controlled trials (RCTs) comparing DOACs with SOC in pediatric patients. Data were synthesized using fixed- or random-effects models to calculate risk ratios (RRs) with 95% confidence intervals (CIs).

**Results:**

Eight RCTs involving 2,002 pediatric patients were included. In terms of efficacy, DOACs were associated with a significant reduction in TE recurrence in treatment studies (*RR* = 0.50; 95% CI 0.25–0.99) and TE occurrence in prophylaxis studies (*RR* = 0.63; 95% CI 0.42–0.95). Regarding safety, major bleeding was comparable to SOC with a favorable trend observed for DOACs (*RR* = 0.64; 95% CI 0.26–1.55). No significant differences were found in all-cause mortality and serious adverse events.

**Conclusion:**

DOACs represent an effective alternative to SOC for the treatment and prevention of thromboembolism in children, demonstrating superior efficacy without increasing the risk of bleeding events.

**Systematic review registration:**

PROSPERO https://www.crd.york.ac.uk/PROSPERO/view/CRD42024506541, CRD42024506541.

## 1 Introduction

Thromboembolic events (TE), encompassing both venous thromboembolism (VTE) and arterial thrombosis, are increasingly recognized complication in pediatric patients, particularly those with chronic medical conditions or with central venous catheters (1, 2). Although VTE represents the predominant manifestation and its incidence in children is lower than that in adults, it has risen significantly in recent decades owing to advances in tertiary care and improved survival of critically ill children (3).

Notably, recent reports from the United States have highlighted a surge in the incidence by more than 200 %, from 34 per 10,000 hospital admissions in 2001 to 106 per 10,000 in 2019 (3, 4). Despite these advances in survival, pediatric TE is associated with substantial morbidity, including the risk of pulmonary embolism and the development of post-thrombotic syndrome, which can significantly impair the quality of life (5). Furthermore, specific pediatric populations, such as those with congenital heart disease (e.g., Fontan circulation) or acute lymphoblastic leukemia, are at a heightened risk of developing these complications (6–8). In these vulnerable groups, effective thromboprophylaxis is crucial for preventing life-threatening events, including stroke and catheter-related thrombosis.

Historically, the standard of care (SOC) for the treatment and prevention of VTE in children has relied on parenteral anticoagulants, such as unfractionated heparin, low-molecular-weight heparin (LMWH), and vitamin K antagonists (VKAs) like warfarin (9, 10). Although effective, these traditional therapies pose significant challenges in the pediatric population. LMWH requires daily subcutaneous injections, which cause pain and anxiety in children and place a substantial burden on caregivers, and they also carry risks of complications such as heparin-induced thrombocytopenia and loss of bone mineral density. Although orally administered, warfarin has a narrow therapeutic index and require frequent coagulation monitoring and complex dosage adjustments (11, 12). Furthermore, achieving stable anticoagulation is often difficult because it is highly susceptible to numerous drug-drug and drug-food interactions (13). Consequently, there is an urgent need for alternative therapeutic options that are effective, safe, and more convenient for pediatric patients.

Direct oral anticoagulants (DOACs), including direct thrombin inhibitors (e.g., dabigatran) and direct factor Xa inhibitors (e.g., rivaroxaban, apixaban, and edoxaban), have revolutionized anticoagulation therapy in adults (14, 15). These agents offer potential advantages over traditional anticoagulants, such as fixed dose regimens, predictable pharmacokinetics, and elimination of routine laboratory monitoring. Notably, in adults with atrial fibrillation or acute VTE, DOACs have demonstrated a superior benefit-risk profile compared with VKAs (16, 17). Building on this success in adults, several large-scale randomized controlled trials (RCTs) have been conducted to evaluate the use of DOACs in the pediatric population, providing high-quality evidence for their clinical utility (18, 19).

Given the emerging data from recent clinical trials, a comprehensive synthesis of evidence is warranted to guide clinical decision making. Although individual studies have reported promising results, an aggregated analysis can provide more precise estimates of treatment effects and safety profiles. Therefore, the aim of this systematic review and meta-analysis was to evaluate the efficacy and safety of DOACs compared with standard anticoagulation therapy for the treatment of acute VTE and the prevention of thromboembolism in high-risk pediatric patients.

## 2 Methods

This systematic review was conducted in accordance with the Preferred Reporting Items for Systematic Reviews and Meta-Analyses (PRISMA) statement. The protocol was registered in the Prospective Register of Systematic Reviews (PROSPERO) (registration number: CRD42024506541).

### 2.1 Data sources and search strategy

We systematically searched PubMed, Embase, Web of Science, and ClinicalTrials.gov for RCTs involving “children” and “direct oral anticoagulant” published up to November 27, 2025. To ensure a comprehensive retrieval of literature, a search strategy combining free-text keywords and controlled vocabulary (e.g., MeSH terms) was used.

### 2.2 Study selection

The eligibility of retrieved records was assessed by two reviewers independently. Initial screening was conducted relied on titles and abstracts to exclude irrelevant studies, followed by a full-text review of potentially eligible articles. Any discrepancies between the reviewers were resolved through discussion or by consulting a third reviewer until a consensus was reached.

Furthermore, to ensure a rigorous study selection, predefined eligibility criteria were applied based on the PICOS framework. Studies were included if they met the following criteria: (1) Population: pediatric patients (aged < 18 years) requiring anticoagulation therapy (for the treatment or prophylaxis of thromboembolic events). (2) Intervention: DOACs, such as rivaroxaban, dabigatran, apixaban, or edoxaban. (3) Comparison: standard of care, such as LMWH, VKAs or placebo. (4) Outcomes: efficacy outcome (e.g., TE recurrence, TE occurrence) and safety outcome (e.g., major bleeding, CRNMB). (5) Study design: RCTs. Conversely, studies were excluded if they met any of the following criteria: duplicate publication, conference abstracts, study protocols, *post-hoc* analyses, single-arm studies, or studies without relevant results.

### 2.3 Data extraction

Each included study was independently reviewed in detail by two reviewers, and relevant details were extracted in a standardized extraction form (registration number, study type, numbers and descriptions of patients enrolled, details of DOAC and comparators, treatment duration, follow-up period, and clinical outcomes). Regarding clinical outcomes, we extracted data for TE recurrence, TE occurrence, all-cause mortality, major bleeding, clinically relevant non-major bleeding (CRNMB), serious adverse events (SAE), and treatment discontinuation due to adverse events (AE) (20). Data were preferentially extracted from peer-reviewed publications, especially in cases of inconsistency, data from ClinicalTrials.gov were cross-referenced.

### 2.4 Quality assessment

The risk of bias in each RCT was assessed using the Cochrane Risk-of-Bias 2 (RoB 2) tool (21). The overall risk of bias was categorized as “low risk,” “some concerns,” or “high risk”. Two reviewers independently appraised eligible articles and any disagreements were resolved through discussion or consultation with a third reviewer.

### 2.5 Statistics analysis

Dichotomous outcomes were expressed as risk ratios (RRs) with 95% confidence intervals (CIs). All statistical analyses were performed using R Studio (version 4.4.2) and the “meta” package. Heterogeneity analysis was assessed using the χ^2^ test (*P*–value) and *I*^2^ statistics. A fixed-effect model was applied when heterogeneity was considered low (*p* < 0.1 and *I*^2^ < 50%). Otherwise, a random-effect model was used. Subgroup analyses were performed based on study purpose (treatment vs. prophylaxis).

### 2.6 Sensitivity and publication bias assessment

A leave-one-out sensitivity analysis was conducted to evaluate the robustness of the results. Contour-enhanced funnel plots were used to assess publication bias in the pooled analyses (22). These tests were conducted when the number of included trials was more than three.

### 2.7 Certainty of evidence assessment

Each pooled result was assessed based on the grading of recommendations assessment, development, and evaluation (GRADE) approach. They were classified into four grades: high, moderate, low, and very low.

## 3 Results

### 3.1 Search results

**Figure 1:**
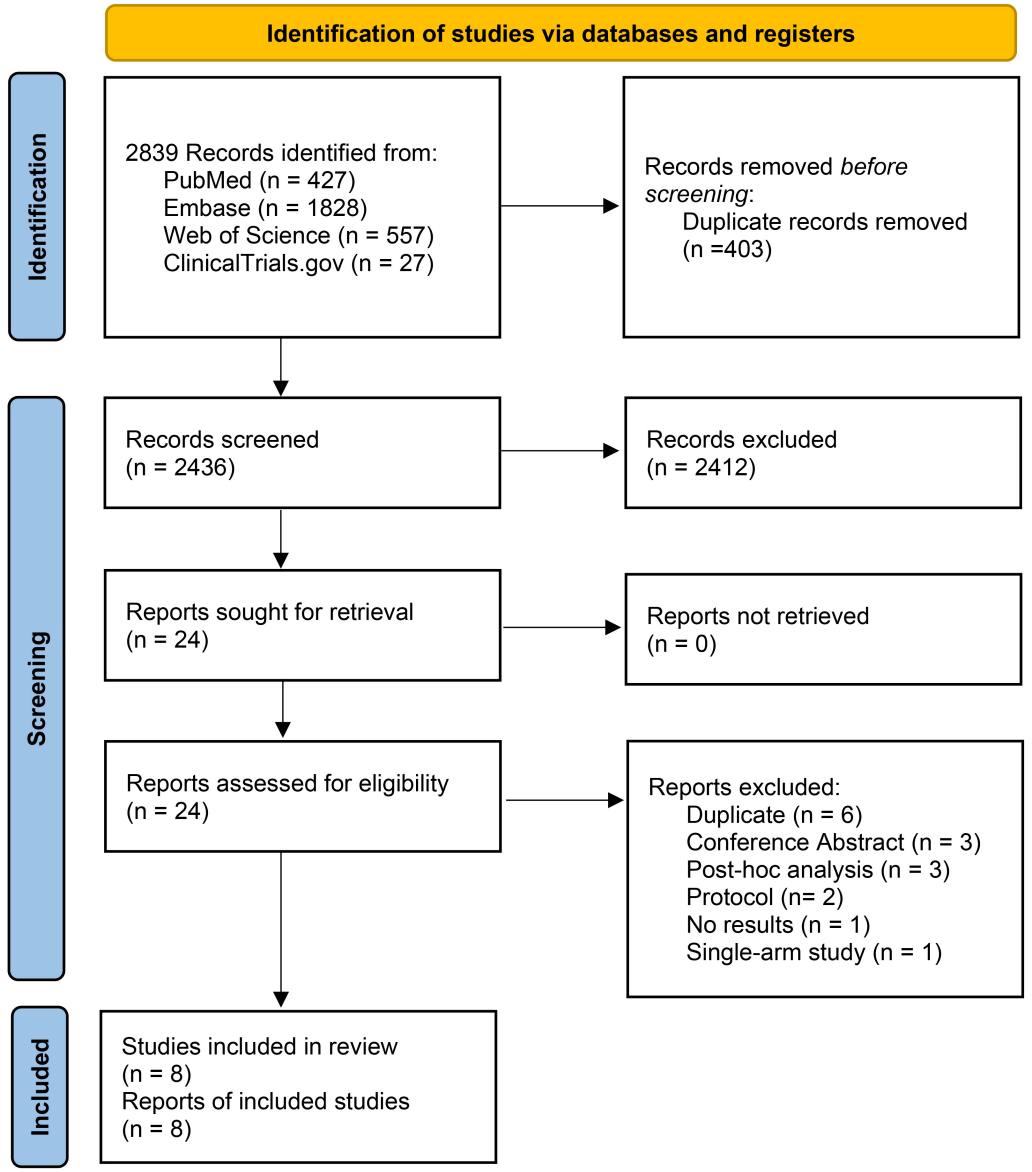
PRISMA flow diagram of the meta-analysis.

A total of 2,839 records were identified through database searches (Supplementary Table S1). After removing 403 duplicate records, 2,436 studies remained for the first screening of titles and abstracts. Of these, 2,412 irrelevant records were excluded. Subsequently, 24 reports were sought for retrieval and all were successfully retrieved and assessed for full-text eligibility. 16 reports were excluded for the following reasons: duplicate publications (*n* = 6), conference abstracts (*n* = 3), *post-hoc* analyses (*n* = 3), protocols (*n* = 2), single-arm study (*n* = 1), no results (*n* = 1). Only eight RCTs were included in systematic review and meta-analysis (18, 19, 23–28) (Figure 1).

### 3.2 Characteristics of included trials

Eight RCTs involving 2002 pediatric patients were included. In terms of study design, all included trials were open-label, multicenter RCTs ranging from phase 2 to phase 4. The included studies were divided equally by indication. Four trials (Eghbali 2020 (23), EINSTEIN-Jr (18), DIVERSITY (19), and NCT02464969 (28)) focused on the treatment of acute VTE or deep vein thrombosis (DVT)/pulmonary embolism (PE). The remaining four trials (UNIVERSE (24), ENNOBLE-ATE (25), Payne 2023 (26), and PREVAPIX-ALL (27)) evaluated thromboprophylaxis in high-risk pediatric populations, prophylactic including patients with congenital heart disease or acute lymphoblastic leukemia (ALL) or lymphoma. Three studies evaluated apixaban, two evaluated dabigatran, two evaluated rivaroxaban, and one evaluated edoxaban. The control groups in the treatment studies predominantly received standard of care (SOC) therapy, consisting of parenteral anticoagulants (e.g., LMWH) and/or vitamin K antagonists (e.g., warfarin). Although the UNIVERSE (24) study compared rivaroxaban with acetylsalicylic acid, and the PREVAPIX-ALL (27) study compared apixaban with no systemic anticoagulation, these comparators represent the prevailing SOC strategies for specific high-risk populations, supporting external validity. The mean age of participants ranged from 3.9 years (UNIVERSE (24)) to 11.7 years (Eghbali 2020 (23)). The proportion of female patients varied from 34.7 to 56.5%. The follow-up duration ranged from one to 13 months. Detailed characteristics of the included studies are summarized in Table 1.

**Table 1:** Characteristics of included trials.

Study name/registry number	Study design	DOAC	Comparator	Treatment duration (months)	Follow-up (months)	Population	Mean age (years)	Female (%)	Purpose	Past history
Eghbali 2020, IRCT20180711040431N1	RCT, phase 3, OL	Dabigatran	enoxaparin or warfarin	6	6	23	11.7	56.5	Treatment	DVT, PE
Male 2020, EINSTEIN-Jr, NCT02234843	RCT, phase 3, OL	Rivaroxaban	unfractionated heparin, LMWH, or VKA	3 or 1^a^	3 or 1^a^	500	9.0	49.0	Treatment	VTE
Halton 2021, DIVERSITY, NCT01895777	RCT, phase 2b/3, OL	Dabigatran	VKA, LMWH, or fondaparinux	3	4	267	11.1	50.2	Treatment	VTE
McCrindle 2021, UNIVERSE, NCT02846532	RCT, phase 3, OL	Rivaroxaban	acetylsalicylic acid	12	13	112	3.9	41.1	Prophylaxis	single-ventricle congenital heart disease and Fontan procedure within 4 months
Portman 2022, ENNOBLE-ATE, NCT03395639	RCT, phase 3, OL	Edoxaban	VKA or LMWH	3	4	167	8.1	34.7	Prophylaxis	cardiac diseases
Payne 2023, NCT02981472	RCT, phase 2, OL	Apixaban	VKA or LMWH	12	12	192	7.8	46.9	Prophylaxis	congenital or acquired heart disease
O'Brien 2024, PREVAPIX-ALL, NCT02369653	RCT, phase 3, OL	Apixaban	no systemic anticoagulation	1	2	512	7.2	43.4	Prophylaxis	acute lymphoblastic leukaemia or lymphoma
NCT02464969	RCT, phase 4, OL	Apixaban	unfractionated heparin, LMWH, or VKA	3.8	7	229	11.3	55.9	Treatment	VTE

RCT, randomized controlled trial; OL, open-labeled; DOAC, direct oral anticoagulant; VKA, vitamin K antagonist; LMWH, low molecular weight heparin; DVT, deep venous thrombosis; PE, pulmonary embolism; VTE, venous thromboembolism.

a In the trial of Male 2020, children younger than 2 years old were treated and followed up for about 1 month.

Patients or population: children.

Intervention: DOACs.

### 3.3 Risk of bias of included trials

The ROB assessment results were summarized in Supplementary Figure S1. All eight trials were judged to have a low overall risk of bias. Although the universal use of an open-label design introduced a potential risk of deviations from intended interventions, this was not considered a significant source of bias due to the application of appropriate intention-to-treat (ITT) analyses. Furthermore, the risk of detection bias was effectively mitigated, as outcome events were objective. The other domains were also assessed as having a low risk of bias, including randomization process, missing outcome data, and selection of the reported result. Consequently, the overall methodological quality of the included evidence was considered high.

### 3.4 Efficacy of the DOACs

**Figure 2:**
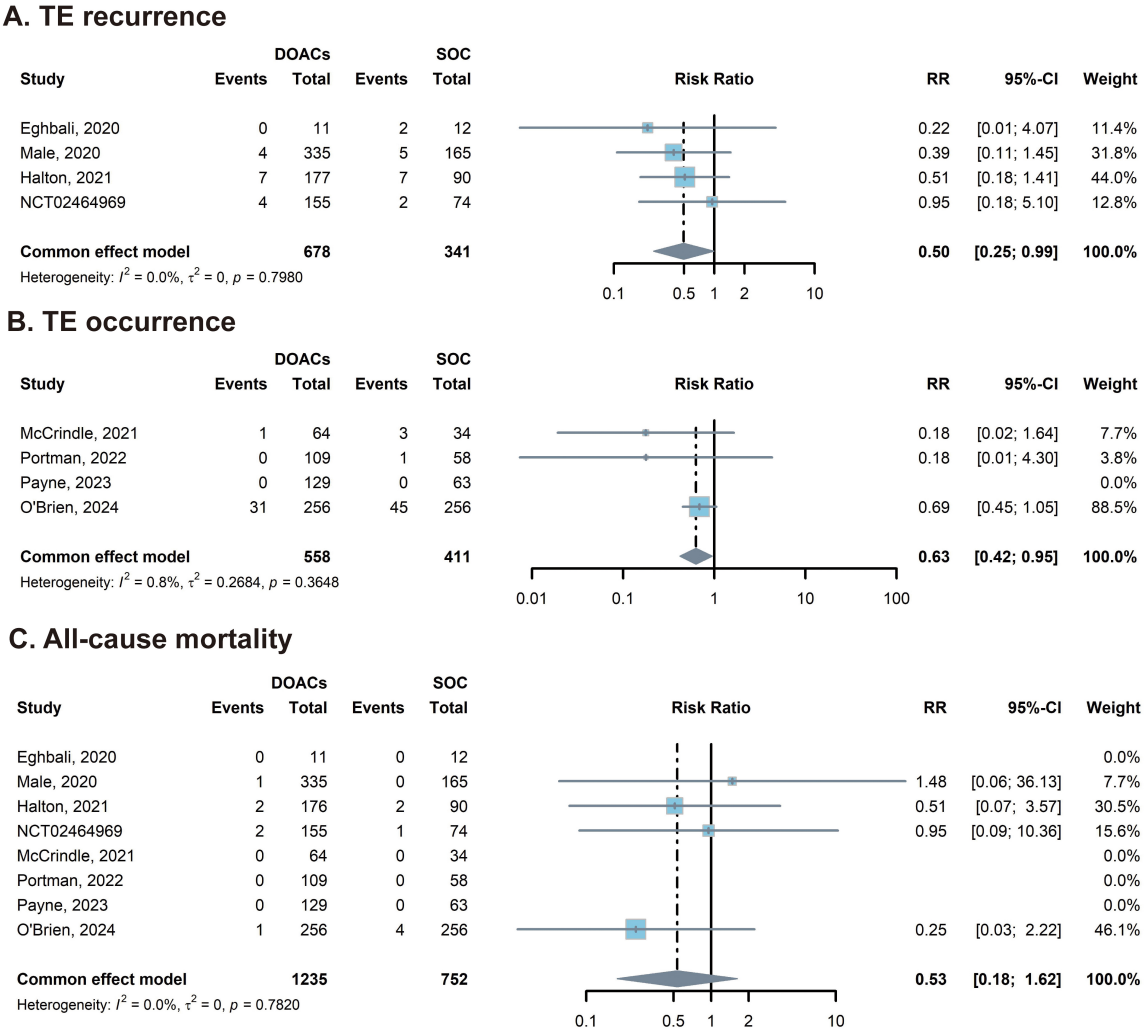
Forest plots of the efficacy. **(A)** The overall analysis of recurrence of TE; **(B)** The overall analysis of occurrence of TE; **(C)** The overall analysis of all-cause mortality. The analyses were performed using a common (fixed) effect model. The blue squares represent the point estimate for each study, and the horizontal lines indicate the 95% confidence intervals (CIs). The diamonds represent the pooled risk ratios (RRs) for the subgroups and the overall population. CI, confidence interval; DOACs, direct oral anticoagulants; RR, risk ratio; SOC, standard of care.

Four trials were included in the analysis of TE recurrence, and the other four trials reported the occurrence of TE in patients with cardiac disease, leukemia, or lymphoma. In the analysis of TE recurrence, the pooled results favored DOACs over the SOC, showing a statistically significant risk reduction (*RR* = 0.50; 95% CI: 0.25–0.99). Similarly, in the analysis of TE occurrence, DOACs demonstrated superior efficacy in preventing thrombotic events compared with the control groups (*RR* = 0.63; 95% CI: 0.42–0.95). In both analyses, heterogeneity was negligible (*I*^2^ = 0% and 0.8%, respectively), suggesting that DOACs are consistently effective in reducing both the recurrence and occurrence of thromboembolism in pediatric patients (Figures 2A, B).

Data on all-cause mortality were available for all eight included trials. Overall, mortality was rare, with four trials reporting zero deaths in both the DOAC and control groups. The pooled analysis demonstrated no statistically significant difference in the risk of all-cause mortality between pediatric patients receiving DOACs and those receiving SOC (*RR* = 0.53; 95% CI: 0.18–1.62). No heterogeneity was observed among the studies (*I*^2^ = 0.0%; *P* = 0.78), suggesting a consistent safety profile for survival across different indications (Figure 2C).

### 3.5 Safety of the DOACs

We considered the following safety outcome measures: major bleeding, CRNMB, SAE, and discontinuation due to AE. Almost all trials reported these safety outcomes, except Eghbali 2020 (23), which did not report CRNMB, SAE, or discontinuation due to AE.

**Figure 3:**
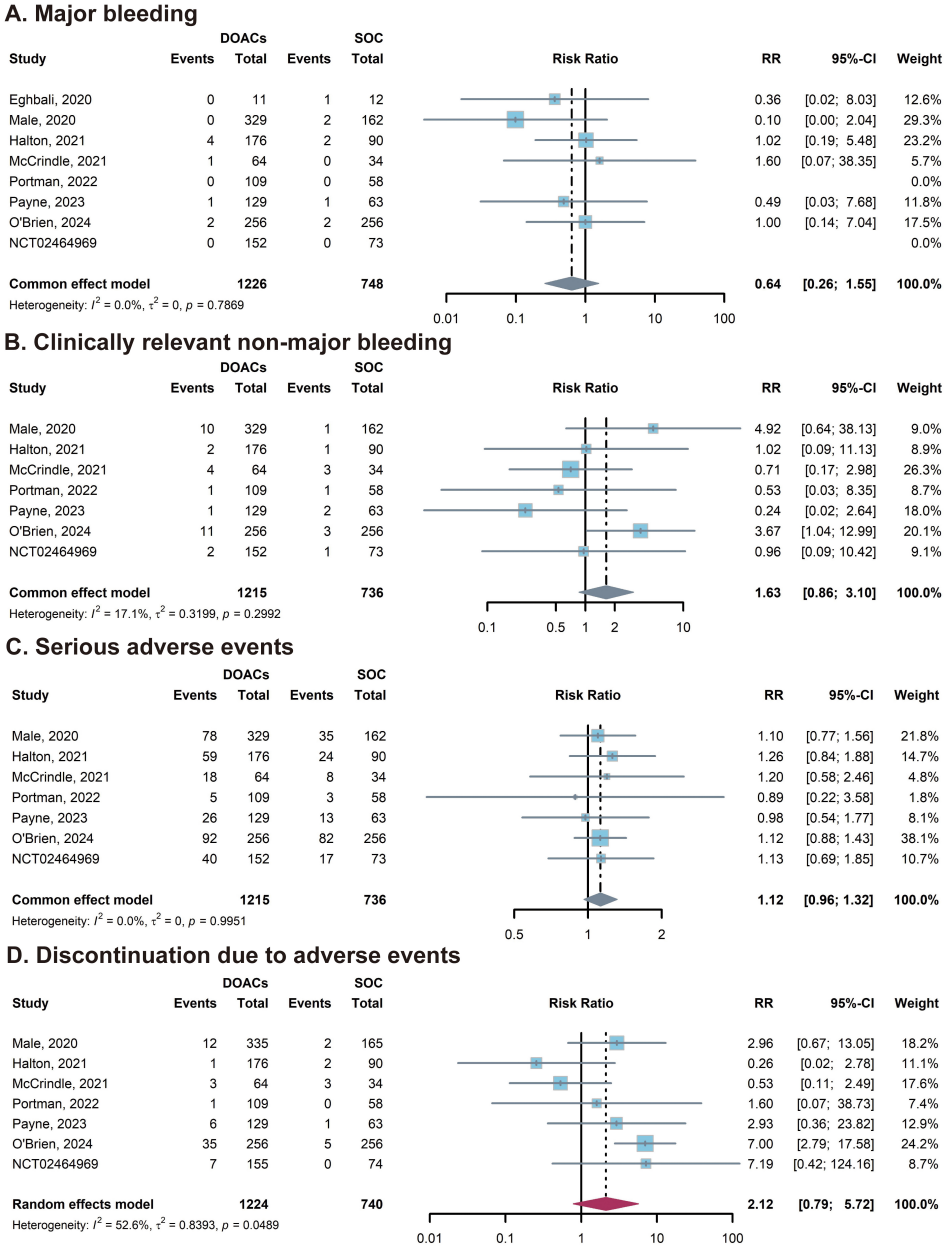
Forest plots of safety outcomes. **(A)** The overall analysis of major bleeding; **(B)** The overall analysis of clinically relevant non-major bleeding; **(C)** The overall analysis of serious adverse events; **(D)** The overall analysis of discontinuation due to adverse events. The blue squares represent the point estimate for each study, and the horizontal lines indicate the 95% confidence intervals (CIs). The diamonds represent the pooled risk ratios (RRs) for the subgroups and the overall population. CI, confidence interval; DOACs, direct oral anticoagulants; RR, risk ratio; SOC, standard of care.

The analysis of major bleeding demonstrated a favorable safety profile for DOACs. The pooled result showed no statistically significant difference between the DOACs and SOC groups, with a RR of 0.64 (95% CI: 0.26–1.55). This suggests that DOACs do not increase the risk of severe, life-threatening hemorrhagic events compared with traditional anticoagulants. Regarding CRNMB, the pooled analysis also indicated no statistically significant difference between the two groups (*RR* = 1.63; 95% CI: 0.86–3.10) (Figures 3A, B).

The risk of SAE was comparable between the treatment arms. The pooled RR was 1.12 (95% CI: 0.96–1.32), indicating that DOACs generally maintain a safety profile similar to SOC regarding overall adverse events. Finally, regarding tolerability, there was no statistically significant difference in the rate of treatment discontinuation due to adverse events (*RR* = 2.12; 95% CI: 0.79–5.72). Although the RR was higher in the DOAC group, the wide confidence interval prevents any definitive conclusion about reduced tolerability (Figures 3C, D).

### 3.6 Subgroup analyses

Subgroup analyses stratified by indication (treatment vs. prophylaxis) were performed to assess potential heterogeneity (Supplementary Figures S2–S3). The analyses revealed consistent safety outcomes across both subgroups, with no statistically significant interactions (*P*_interaction_ > 0.05 for all endpoints). Specifically, the risk estimates for major bleeding were similar between the treatment (*RR* = 0.48; 95% CI: 0.15–1.58) and prophylaxis subgroups (*RR* = 0.93; 95% CI: 0.23–3.75; *P*_interaction_ = 0.48). Similarly, no significant subgroup differences were observed for CRNMB (*P*_interaction_ = 0.49), all-cause mortality (*P*_interaction_ = 0.38), SAE (*P*_interaction_ = 0.76), and discontinuation due to AE (*P*_interaction_ = 0.81). These findings suggest that the safety profile of DOACs remains stable regardless of the indication.

### 3.7 Sensitivity analyses

The results of all-cause mortality, major bleeding, CRNMB, and SAE were robust. The exclusion of any single study did not significantly alter the direction of the effect size or magnitude of heterogeneity, confirming the stability of these findings (Supplementary Table S2).

However, variability was observed in the other outcomes. The significant reduction in TE recurrence was lost when the trials of Eghbali 2020 (23), EINSTEIN-Jr (18), or DIVERSITY (19) were omitted. Similarly, the significant benefit for TE occurrence became non-significant after excluding the study by UNIVERSE (24) or PREVAPIX-ALL (27). Regarding safety, for discontinuation due to AE, the initial non-significant result became statistically significant, with an increased risk associated with DOACs when excluding the study by DIVERSITY (19) (*RR* = 2.81; 95% CI: 1.08–7.31) or UNIVERSE (24) (*RR* = 3.09; 95% CI: 1.21–7.85).

### 3.8 Publication bias

Publication bias was assessed using contour-enhanced funnel plots and the trim-and-fill method (Supplementary Figure S4). The funnel plot for major bleeding (Supplementary Figure S4D) revealed a symmetrical distribution of the studies, suggesting no evidence of publication bias for this primary safety outcome. However, asymmetry was observed in other outcomes. The trim-and-fill method was applied to adjust for this asymmetry by imputing hypothetical missing studies (represented as hollow circles), which predominantly fell within the white regions corresponding to non-significance (*P* > 0.10). This pattern strongly suggests that the asymmetry was likely driven by the absence of small studies reporting negative or null finding.

Following the trim-and-fill adjustment, the pooled RRs for recurrence (0.53; 95% CI: 0.27–1.05) and occurrence (0.69; 95% CI: 0.46–1.03) were altered which shifted from statistically significant to non-significant. For discontinuation due to AE, the adjusted result (*RR* = 4.90; 95% CI: 1.50–15.95) indicated a significant increase in risk. These shifts indicate that publication bias may have influenced both the magnitude and statistical significance of the reported effect estimates.

### 3.9 Quality of evidence

The certainty of evidence was assessed using the GRADE approach. Overall, the quality of evidence across outcomes ranged from moderate to high (Table 2). The certainty of evidence for recurrence and occurrence was graded as moderate, reflecting concerns that trim-and-fill analyses indicated missing studies with negative results may alter statistical significance. The evidence for SAE was graded as high, indicating that further research is unlikely to change our confidence in estimating the effect. For major bleeding, CRNMB, and discontinuation due to AE, the evidence was graded as moderate because of serious imprecision, as the small number of events, produced wide CIs, suggesting possible benefit or harm.

**Table 2:** Summary of findings (SoF) table assessing the quality of evidence according to GRADE methodology.

Outcomes	Illustrative comparative risks^*^ (95% CI)		Relative effect (95% CI)	No of participants (studies)	Quality of the evidence (GRADE)	Comments
	Assumed risk	Corresponding risk				
	SOC	DOACs				
Recurrence	47 per 1,000	23 per 1,000 (12–46)	RR 0.50 (0.25–0.99)	1,019 (4 studies)	⊕⊕⊕⊖**moderate**^**1**^	–
Occurrence	119 per 1,000	75 per 1,000 (50–113)	RR 0.63 (0.42–0.95)	969 (4 studies)	⊕⊕⊕⊖**moderate**^**1**^	–
All-cause mortality	9 per 1,000	5 per 1,000 (2–15)	RR 0.53 (0.18–1.62)	1,987 (8 studies)	⊕⊕⊕⊖**moderate**^**2**^	–
Major bleeding	11 per 1,000	7 per 1,000 (3–17)	RR 0.64 (0.26–1.55)	1,974 (8 studies)	⊕⊕⊕⊖**moderate**^**2**^	–
CRNMB	16 per 1,000	27 per 1,000 (14–51)	RR 1.63 (0.86–3.1)	1,951 (7 studies)	⊕⊕⊕⊖**moderate**^**2**^	–
SAE	247 per 1,000	277 per 1,000 (237–326)	RR 1.12 (0.96–1.32)	1,951 (7 studies)	⊕⊕⊕⊕**high**	–
Discontinuation due to AE	18 per 1,000	37 per 1,000 (14 to 100)	RR 2.12 (0.79 to 5.72)	1,964 (7 studies)	⊕⊕⊕⊖**moderate**^**2**^	–

* The basis for the **assumed risk** (e.g. the median control group risk across studies) is provided in footnotes. The **corresponding risk** (and its 95% confidence interval) is based on the assumed risk in the comparison group and the **relative effect** of the intervention (and its 95% CI).

CI, Confidence interval; RR, Risk ratio; CRNMB, clinically relevant non-major bleeding; SAE, serious adverse event.

GRADE Working Group grades of evidence **High quality:** further research is very unlikely to change our confidence in the estimate of effect. **Moderate quality:** further research is likely to have an important impact on our confidence in the estimate of effect and may change the estimate. **Low quality:** further research is very likely to have an important impact on our confidence in the estimate of effect and is likely to change the estimate. **Very low quality:** we are very uncertain about the estimate.

1 Suspected publication bias: the trim-and-fill analysis suggested asymmetry in the funnel plot, and the statistical significance was lost after adjustment.

2 Serious imprecision: downgraded due to wide confidence intervals crossing the line of no effect and/or low number of events.

## 4 Discussion

### 4.1 Summary of main findings

This systematic review and meta-analysis, incorporating data from eight large-scale RCTs and over 2,000 pediatric patients, provides comprehensive evidence regarding the use of DOACs for both the treatment and prevention of TE. Our principal finding is that DOACs demonstrate a superior benefit-risk profile compared to SOC. In terms of efficacy, DOACs were associated with a significant reduction in the TE recurrence in treatment settings and TE occurrence in prophylaxis settings. Regarding safety, DOACs showed a comparable risk to SOC across major bleeding and CRNMB. Subgroup analyses confirmed that the efficacy and safety signals were consistent across different clinical indications and dosing intensities.

The demonstration of superior prophylactic efficacy in our pooled analysis is particularly noteworthy. Unlike treatment trials in which DOACs were compared against therapeutic doses of potent anticoagulants (LMWH/VKA), prophylaxis trials often compared DOACs against less potent agents (e.g., aspirin in UNIVERSE (24)) or with no systemic anticoagulation (e.g., PREVAPIX-ALL (27)). However, it is important to note that neither of these individual trials achieved statistical significance for thromboprotection on its own. Consequently, our findings highlight that for high-risk pediatric populations, such as those with Fontan circulation or acute leukemia, active anticoagulation with DOACs may provide significantly better thromboprotection than the previous standard of conservative management or antiplatelet therapy, though these pooled results should be interpreted with caution.

Importantly, our analysis showed no significant difference in major bleeding and CRNMB which necessitate medical intervention or hospitalization. A favorable trend toward reduced major bleeding was observed in DOACs (*RR* = 0.64; 95% CI 0.26–1.55), reinforcing the potential of these agents to mitigate severe hemorrhagic risks. Regarding CRNMB, the primary analysis suggested a trend toward increased risk (*RR* = 1.63). However, sensitivity analysis revealed that the trend was largely driven by a single outlier study (PREVAPIX-ALL) (27). Excluding this trial markedly attenuated the risk estimate to 1.12 (95% CI 0.52–2.44), a value approaching unity. The residual slight elevation is clinically negligible and mechanistically anticipated. Moreover, unlike parenteral anticoagulants, oral agents may exert a local anticoagulant effect within the intestinal lumen prior to absorption. This mechanism is associated with a slightly higher risk of mucosal bleeding (e.g., epistaxis and gingival bleeding), which was classified as CRNMB. Nevertheless, the elevated CRNMB signal observed in the PREVAPIX-ALL warrants specific contextualization. First, the comparator arm in the study received no anticoagulation rather than active anticoagulants (e.g., LMWH or VKA), rendering a numerical increase in bleeding pharmacologically expected. Second, the study population consisted of patients with leukemia, a group uniquely vulnerable to chemotherapy-induced mucositis and potential thrombocytopenia. These factors compromise mucosal integrity, predisposing children (particularly those under ten years old) to localized events such as epistaxis. Therefore, the results suggest that while DOACs maintain a major safety advantage, they possess a comparable CRNMB profile to standard anticoagulants. Clinicians should weigh the bleeding risk of the individual patient against the benefits in efficacy and practical advantages of an injection-free regimen (29).

Beyond efficacy, the practical dimensions of safety and adherence are paramount, particularly in young children (30, 31). However, it is crucial to note that children below 2 years of age (particularly neonates and infants) were scarcely represented in the available pediatric DOAC studies. Therefore, clinicians must exercise great caution when using DOACs in this highly sensitive population until more robust efficacy and safety data become available. Traditional anticoagulants (LMWH and VKAs) pose significant challenges: LMWH necessitates painful daily subcutaneous injections, while VKAs require frequent venipunctures for INR monitoring and complex dosage adjustments. These burdens not only compromise pediatric adherence but also impose substantial healthcare costs (5). In our meta-analysis, the incidence of SAE was statistically comparable to SOC. While a slight numerical increase in the RR was observed, this is likely driven by background morbidity, such as disease progression (e.g., recurrent thrombosis) or chemotherapy-related toxicity (e.g., febrile neutropenia) rather than direct anticoagulant effects. Regarding tolerability, the primary analysis suggested a potential increase in discontinuation due to AE with DOACs (*RR* = 2.12; 95% CI 0.79–5.72) with moderate heterogeneity (*I*^2^ = 52.6%). However, sensitivity analysis identified the study by PREVAPIX-ALL as the primary source of this variance. Excluding this trial reduced the heterogeneity substantially to 19.1% and attenuated the risk ratio to 1.43 (95% CI 0.55–3.74). The disparity appears methodological rather than biological. The PREVAPIX-ALL trial compared apixaban with no anticoagulation, creating a structural imbalance where “discontinuation due to drug-related events” was inherently improbable. Furthermore, minor mucosal bleeding events often necessitated protocol-mandated treatment interruption in the DOAC arm. When compared to active anticoagulants in other trials, DOAC discontinuation rate remained statistically indistinguishable from the SOC.

Notably, health economic analyses in adults have consistently demonstrated that DOACs are cost-saving compared to SOC, largely driven by the reduction in monitoring costs, hospitalization time, and administration complexity (32, 33). DOACs offer a similarly compelling value proposition in the pediatric setting. With their oral administration and fixed-dosing regimens supported by pharmacokinetic/pharmacodynamic (PK/PD) studies, DOACs obviate the trauma of injections and the burden of monitoring (34, 35). This shift represents a significant improvement in the quality of life for children and their caregivers, potentially translating into superior real-world adherence and reducing long-term healthcare resource utilization.

Our findings expand the knowledge gained from previous meta-analyses (36, 37). While earlier reviews indicated that DOACs were superior to SOC in preventing TE recurrence but showed no difference in thromboprophylaxis, our study provides updated evidence. By incorporating newly published RCTs into both subgroups, we increased statistical power and demonstrated superior efficacy for DOACs in prophylaxis for the first time. The overall risk of bias and heterogeneity of the included RCTs were low, which strengthen the validity of our results. Although the GRADE assessment indicated moderate certainty for most outcomes owing to serious imprecision, this should be interpreted cautiously. Importantly, the observed imprecision reflects the very low number of severe events (e.g., all-cause mortality and major bleeding) in both groups, which should be viewed as a positive reflection of the overall safety of antithrombotic therapy in children rather than a limitation of study design. Furthermore, our findings are supported by emerging observational data, and long-term extension studies provide complementary evidence (38). For instance, the post-study surveillance of the EINSTEIN-Jr (18) program demonstrated that during extended anticoagulation, the rates of recurrent VTE and major bleeding remained low, consistent with the long-term safety profile observed in adults (39). This provides reliable clinical evidence supporting the use of DOACs for extended therapy in children. Similarly, a prospective single-arm cohort study of dabigatran indicated a favorable safety profile for secondary VTE prevention (40). Furthermore, considering that obesity is an independent risk factor for pediatric VTE (41), a *post-hoc* analysis of the PREVAPIX-ALL (27) trial suggested that DOACs maintain their preventive efficacy in obese and pediatric patients, thereby addressing a critical need for this specific high-risk subgroup (42).

While DOACs provide a pivotal alternative for pediatric thrombosis, currently only specific agents (rivaroxaban and dabigatran) have gained regulatory approval from Health Canada, the FDA, and the EMA (see Supplementary Table S3) (43). Despite these benefits, strict eligibility criteria must be applied in clinical decision-making. Based on the PK/PD profiles of these agents, DOACs should be reserved for hemodynamically stable patients with preserved hepatic and renal function. Specific contraindications include the use of DOACs in patients with triple-positive antiphospholipid syndrome (APS) for whom warfarin remains the standard therapy (44). Additionally, dosing must be precisely weight-adjusted (e.g., for rivaroxaban) (45, 46), and caution is needed in patients with severe gastrointestinal complications (e.g., short bowel syndrome) that may impair absorption (47). Finally, implementation challenges persist. Safety concerns persist regarding the management of overdose or uncontrolled bleeding, given the limited availability of specific reversal agents for the pediatric population (5). Recent literature has proposed useful management strategies for DOAC overdose in children to provide valuable guidance for clinical practice (48). In low- and middle-income countries (LMICs), broader adoption of DOACs is hindered by technical barriers, including limited specialized experience, insufficient monitoring resources, and substantial cost constraints (49).

### 4.2 Limitations

The results of this meta-analysis should be interpreted in light of several limitations. First, although our primary analyses showed robust results, potential publication bias remains a concern where small negative studies may have remained unpublished. Second, all included trials utilized an open-label design, which may have introduced performance bias, although the use of blinded central adjudication was used for the outcomes. Third, the total number of major safety events was small, resulting in wide confidence intervals. Fourth, there was a lack of detailed and consistent reporting on D-dimer levels across the included publications, which limited further evaluation of the potential correlation between this biomarker and the clinical efficacy or safety of DOACs in the pediatric population. Therefore, although the overall trend favored DOACs, these findings should be interpreted with caution.

## 5 Conclusion

In conclusion, DOACs demonstrate a superior alternative compared to SOC for pediatric thromboembolism in the treatment and prophylaxis, with a comparable safety profile. Although a potential trend toward minor bleeding exists, the risk is clinically manageable and outweighed by the substantial benefits of an injection-free regimen, improved cost-effectiveness, and enhanced quality of life. Therefore, DOACs represent a transformative therapeutic option for pediatric thromboses. Nevertheless, their use requires certain patient selection based on renal function and underlying conditions. Ongoing pharmacovigilance is warranted to monitor long-term safety, particularly in neonates and infants.

## Data Availability

The original contributions presented in the study are included in the article/supplementary material, further inquiries can be directed to the corresponding authors.
