# Supplementary material for: Efficacy and safety of direct oral anticoagulants in pediatric patients: a systematic review and meta-analysis
Source: Front Public Health. 2026 Apr 7;14:1787819. doi: 10.3389/fpubh.2026.1787819 (PMC13096052; doi:10.3389/fpubh.2026.1787819)
Supplement: Supplementary file 1 [file Supplementary_File_1.docx]

Supplementary Material

**Supplementary Table S1.** Search strategies

| Database | Search Query / String | Results |
| --- | --- | --- |
| Pubmed | ("Pediatrics"[MeSH] OR "pediatric*"[Title/Abstract] OR "Child"[MeSH] OR "child*"[Title/Abstract]) AND (("DOAC*"[Title/Abstract] OR "direct oral anticoagulant*"[Title/Abstract] OR "non vitamin k antagonist*"[Title/Abstract] OR " VKA"[Title/Abstract] ) OR ("Rivaroxaban"[MeSH] OR "rivaroxaban "[Title/Abstract] OR "xarelto"[Title/Abstract]) OR ("apixaban"[Supplementary Concept] OR "apixaban"[Title/Abstract] OR "eliquis"[Title/Abstract]) OR ("edoxaban"[Supplementary Concept] OR "edoxaban"[Title/Abstract] OR "lixiana"[Title/Abstract]) OR ("Dabigatran"[MeSH] OR "dabigatran"[Title/Abstract] OR "pradaxa"[Title/Abstract])) | 427 |
| Embase | ('pediatrics'/exp OR 'pediatric*':ti,ab,kw OR 'child'/exp OR 'child*':ti,ab,kw) AND (('doac*':ti,ab,kw OR 'direct oral anticoagulant*':ti,ab,kw OR 'non vitamin k antagonist*':ti,ab,kw OR 'vka':ti,ab,kw) OR ('rivaroxaban'/exp OR 'rivaroxaban':ti,ab,kw OR 'xarelto':ti,ab,kw) OR ('apixaban' OR 'apixaban':ti,ab,kw OR 'eliquis':ti,ab,kw) OR ('edoxaban' OR 'edoxaban':ti,ab,kw OR 'lixiana':ti,ab,kw) OR ('dabigatran'/exp OR 'dabigatran':ti,ab,kw OR 'prazaxa':ti,ab,kw)) | 1828 |
| Web of Science | TS=(pediatric* OR paediatric* OR child* OR infant* OR adolescent* OR neonat*)  AND TS=("doac*" OR "direct oral anticoagulant*" OR "novel oral anticoagulant*" OR "noac*" OR "non-vitamin k antagonist*" OR "rivaroxaban" OR "xarelto" OR "apixaban" OR "eliquids" OR "edoxaban" OR "liliana" OR "savasa" OR "dabigatran" OR "pradaxa") | 557 |

**Supplementary Table S2.** Summary of leave-one-out sensitivity analysis for efficacy and safety outcomes.

| Outcome | Original Pooled RR (95% CI) | Robustness | Influential Study (Excluded) | Re-calculated RR (95% CI) | Change in Significance |
| --- | --- | --- | --- | --- | --- |
| recurrence | 0.50 (0.25-0.99) | Sensitive | Eghbali et al. | 0.53 (0.26-1.09) | Sig. to Non-sig. |
|  |  |  | Male et al. | 0.54 (0.24-1.23) |  |
|  |  |  | Halton et al. | 0.49 (0.19-1.25) |  |
| occurrence | 0.63 (0.42-0.95) | Sensitive | McCrindle et al. | 0.67 (0.44-1.01) | Sig. to Non-sig. |
|  |  |  | O’Brien et al. | 0.18 (0.03-1.10) |  |
| All-cause mortality | 0.53 (0.18-1.62) | Robust | None | N/A | No change |
| Major bleeding | 0.64 (0.26-1.55) | Robust | None | N/A | No change |
| Clinically relevant non-major bleeding | 1.63 (0.86-3.10) | Robust | None | N/A | No change |
| Serious adverse events | 1.12 (0.96-1.32) | Robust | None | N/A | No change |
| Discontinuation due to adverse events | 2.12 (0.79-5.72) | Sensitive | Halton et al. | 2.81 (1.08-7.31) | Non-sig. to Sig. |
|  |  |  | McCrindle et al. | 3.09 (1.21-7.85) |  |

**Supplementary Table S3**. Current approval status of DOACs for pediatric patients.

| Drugs | US FDA Approval Status | European EMA Approval Status | Health Canada Approval Status | Main Pediatric Indications |
| --- | --- | --- | --- | --- |
| Rivaroxaban/Xarelto | Approved | Approved | Approved | Treatment of VTE and prevention of recurrent VTE  Thromboprophylaxis after Fontan procedure (FDA/Health Canada specific ages) |
| Dabigatran/Pradaxa | Approved | Approved | Not Approved | Treatment of VTE and prevention of recurrent VTE (only FDA and EMA) |
| Apixaban/Eliquis | Not Approved | Approved | Not Approved | Treatment of VTE (only EMA) |
| Edoxaban/Lixiana | Not Approved | Not Approved | Not Approved | Under investigation in pediatric clinical trials |

Abbreviations: VTE, venous thromboembolism.

**
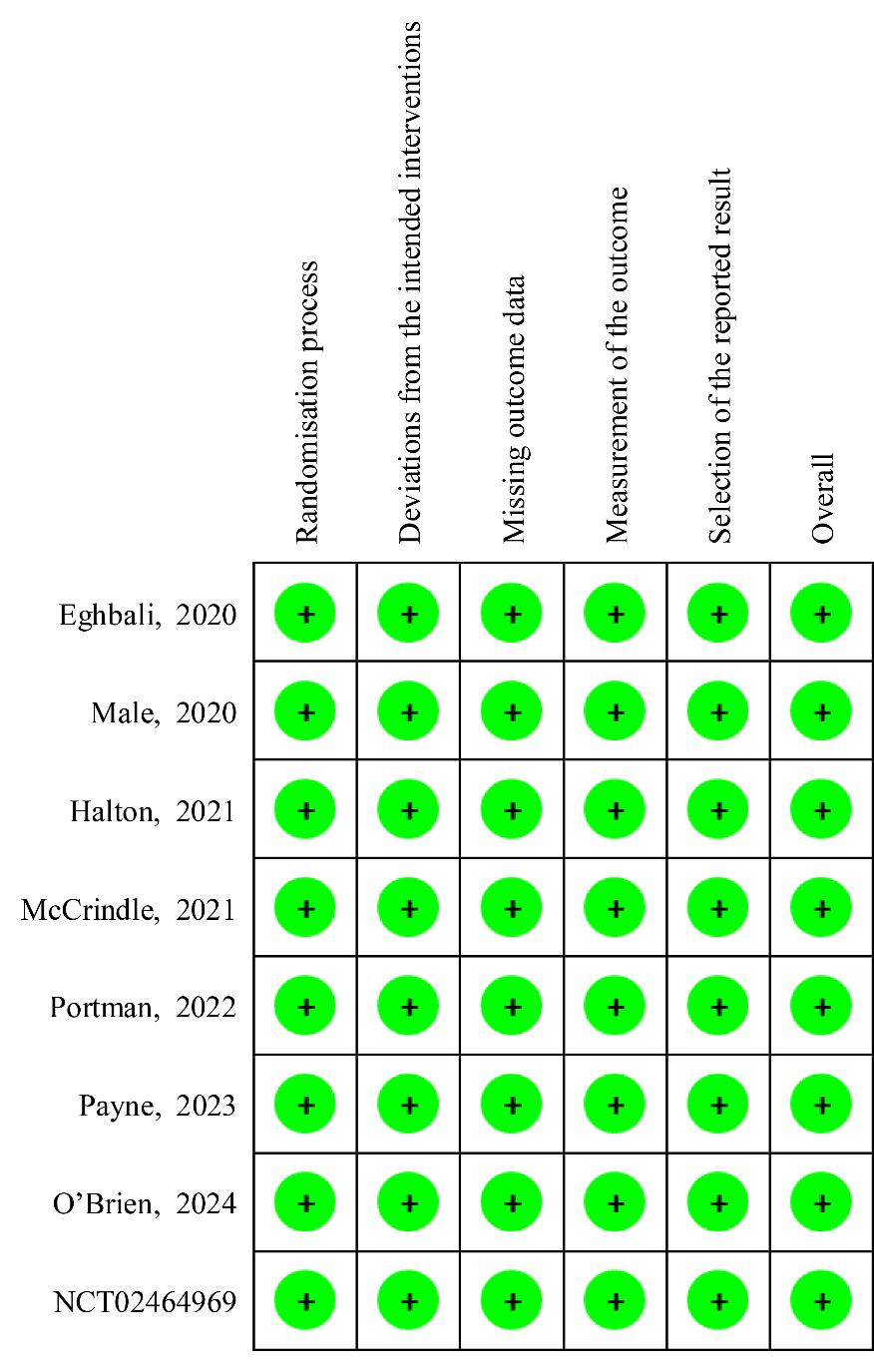
**

**Supplementary Figure S1.** Risk of Bias summary for included randomized controlled trials. Green circles with a plus sign (+) indicate a low risk of bias. Note: Studies without a primary publication are identified by their ClinicalTrials.gov registry number (e.g., NCT02464969).


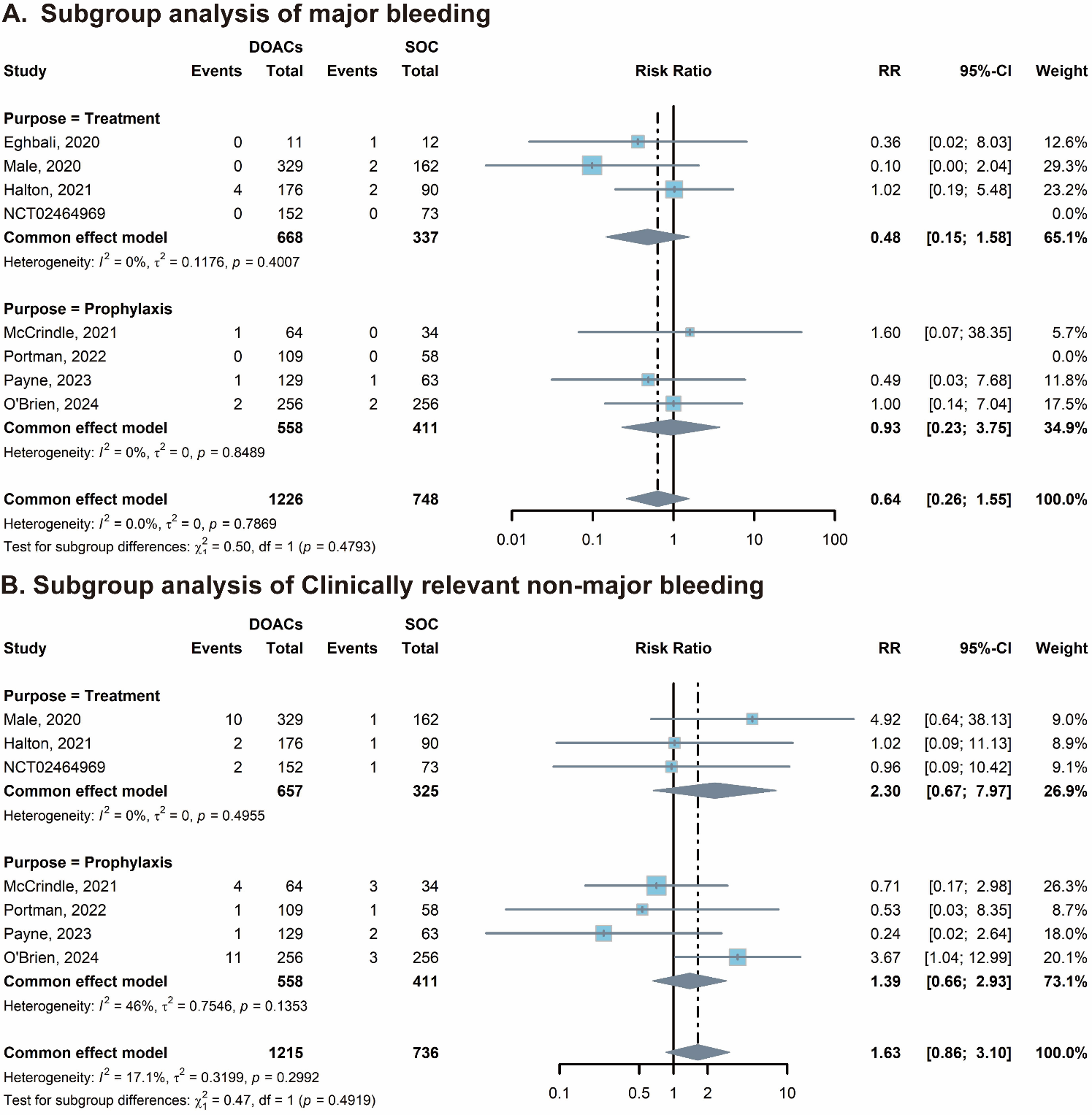


**Supplementary Figure S2.** Forest plots of subgroup analyses for bleeding outcomes stratified by study indication (Treatment vs. Prophylaxis). (A) Subgroup analysis of major bleeding; (B) Subgroup analysis of clinically relevant non-major bleeding. The analyses were performed using a common (fixed) effect model. The blue squares represent the point estimate for each study, and the horizontal lines indicate the 95% confidence intervals (CIs). The diamonds represent the pooled risk ratios (RRs) for the subgroups and the overall population. Abbreviations: CI, confidence interval; DOACs, direct oral anticoagulants; RR, risk ratio; SOC, standard of care.


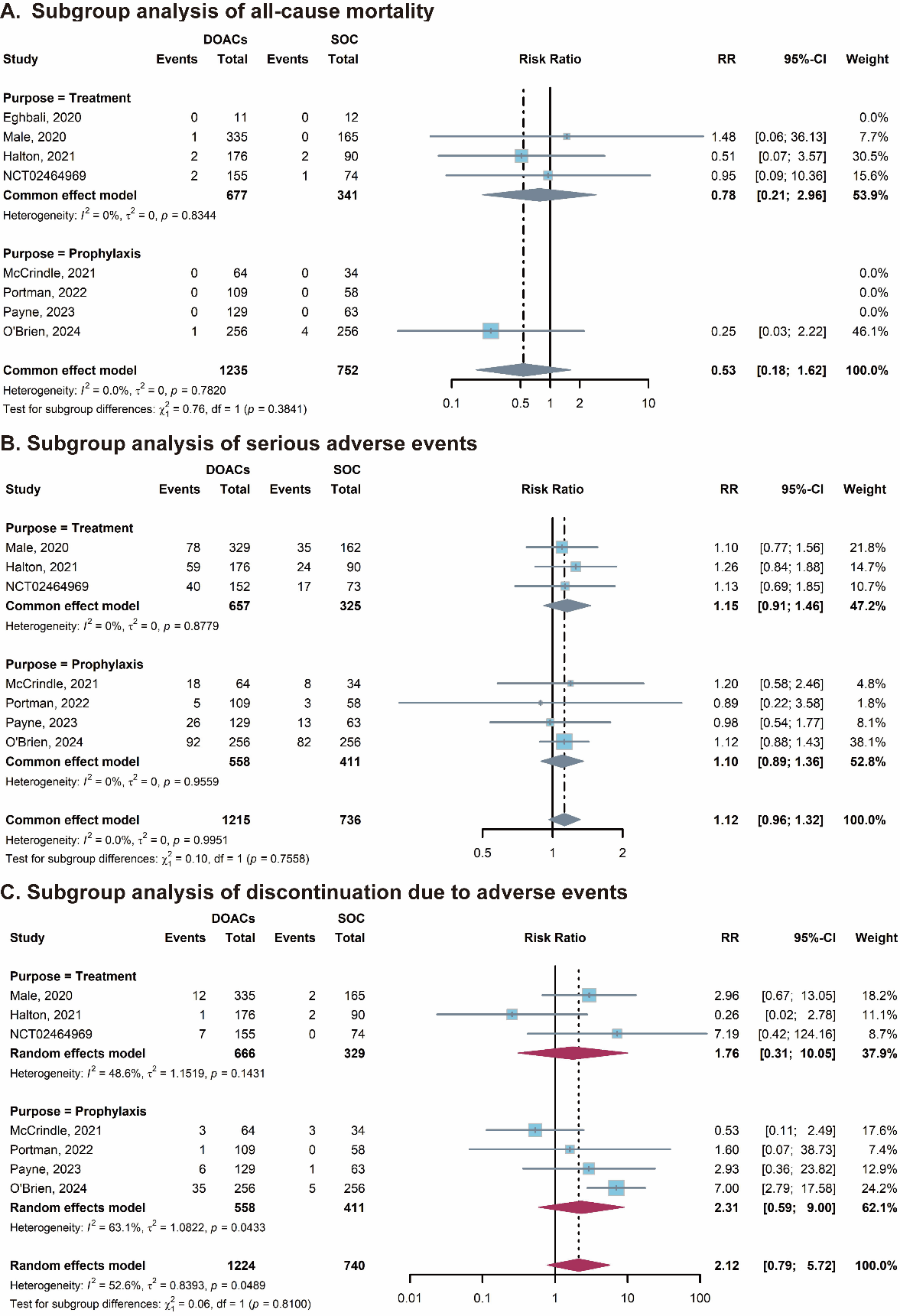


**Supplementary Figure S3.** Forest plots of subgroup analyses for general safety outcomes stratified by study indication (Treatment vs. Prophylaxis). (A) Subgroup analysis of all-cause mortality; (B) Subgroup analysis of serious adverse events; (C) Subgroup analysis of discontinuation due to adverse events. The blue squares represent the point estimate for each study, and the horizontal lines indicate the 95% confidence intervals (CIs). The diamonds represent the pooled risk ratios (RRs) for the subgroups and the overall population. Abbreviations: CI, confidence interval; DOACs, direct oral anticoagulants; RR, risk ratio; SOC, standard of care.


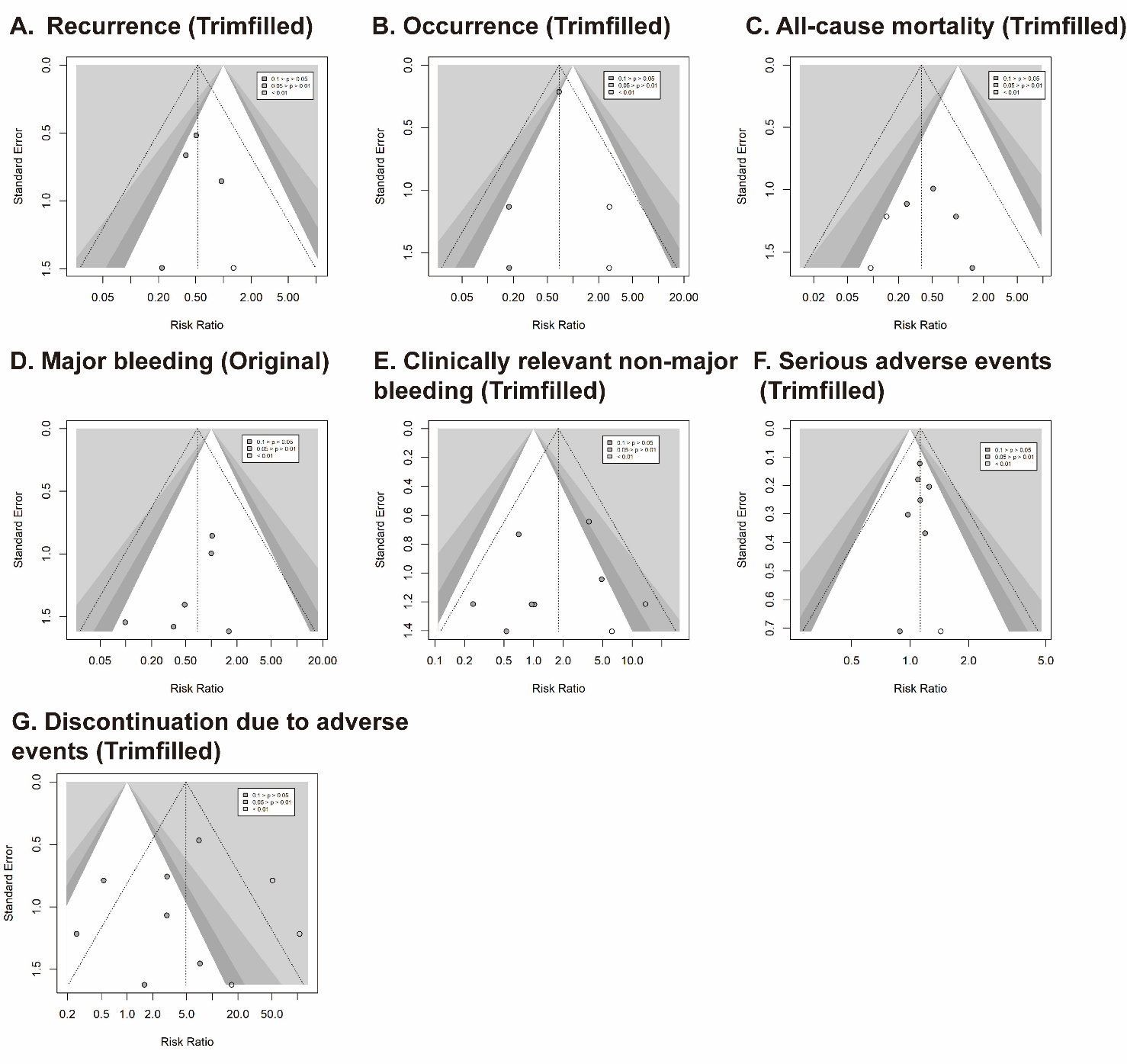


**Supplementary Figure S4.** Assessment of publication bias using contour-enhanced funnel plots. (A) TE recurrence (Trim-and-fill adjusted); (B) TE occurrence (Trim-and-fill adjusted); (C) All-cause mortality (Trim-and-fill adjusted); (D) Major bleeding (Original unadjusted plot); (E) Clinically relevant non-major bleeding (Trim-and-fill adjusted); (F) Serious adverse events (Trim-and-fill adjusted); (G) Discontinuation due to adverse events (Trim-and-fill adjusted). The vertical axis represents the standard error (study precision), and the horizontal axis represents the risk ratio (RR) on a logarithmic scale. The shaded regions correspond to levels of statistical significance: the white area indicates non-significance (*P* > 0.10), the light gray area indicates 0.05 < *P* < 0.10, and the dark gray areas indicate statistical significance (*P* < 0.05). Gray filled circles represent the observed studies included in the meta-analysis. Hollow circles represent hypothetical missing studies imputed by the trim-and-fill method to adjust for funnel plot asymmetry.
